# Patterns of instrumental activities of daily living between community-dwelling older adults

**DOI:** 10.1590/1980-57642021dn15-030009

**Published:** 2021

**Authors:** Antonia de Azevedo Falcão Sigrist, Ana Claudia Becattini Oliveira, Helenice Charchat Fichman

**Affiliations:** 1 Psychology Department, Pontifícia Universidade Católica do Rio de Janeiro – Rio de Janeiro, RJ, Brazil.; 2 Clinical Medicine Department, School of Medicine, Universidade de São Paulo – São Paulo, SP, Brazil.

**Keywords:** population heterogeneity, elderly, clustering, functional performance, características da população, idoso, análise por conglomerados, desempenho funcional

## Abstract

The ability to perform instrumental activities of daily living (IADLs) is an important marker within the aging process, as it reflects the level of independence to perform in the real world. However, there is a scarcity of studies that investigate the different performance profiles among older adults.

**Objective:**

To investigate the heterogeneity present in the community of elderly people, grouping them based on characteristics and patterns observed through an objective performance-based assessment.

**Methods:**

Participants were selected according to the following inclusion criteria: fluency in Portuguese, ^3^60 years, regular participation in a social program offered by the government of Rio de Janeiro, and absence of a caregiver. The evaluation of IADLs was determined by the total and brief version of the University of California, San Diego Performance-Based Skills Assessment (UPSA) and the Lawton and Brody IADL scale. The Brief Cognitive Screening Battery and the Mini-Mental State Examination were used to characterize the sample, in addition to the Geriatric Depression Scale. A total of 61 elderly people with an average age of 72.5 years, predominantly females (85.2%), and average education of 11.2 were evaluated and grouped according to their performance at UPSA through clustering analysis.

**Results:**

The analysis revealed three grouping patterns, subdividing the sample into subgroups that differed significantly in terms of age, education, global cognition, and all instrumental activities assessed by UPSA — planning, finance, communication, transportation, and household chores.

**Conclusions:**

This study was able to identify the heterogeneity present between the elderly people in the different factors that compose the IADLs through a performance-based assessment.

## RESUMO

A capacidade para realização das atividades instrumentais da vida diária (AIVDs) é um importante marcador no envelhecimento, uma vez que reflete o grau de independência para atuação no mundo real. No entanto, há uma escassez de estudos que se proponham a investigar os diferentes perfis de desempenho entre idosos.

**Objetivo:**

Investigar a heterogeneidade presente em idosos da comunidade, agrupando-os a partir de características e padrões observados através de uma avaliação objetiva baseada em desempenho.

**Métodos:**

Participantes foram selecionados conforme critérios de inclusão: ter fluência no Português; ter 60 ou mais anos; participar de forma regular no programa de casas de convivência do Rio de Janeiro e não possuir cuidador. A avaliação das AIVDs foi determinada pela versão total e breve do *The University of California, San Diego Performance-Based Skills Assessment* (UPSA) e pela escala AIVD de Lawton e Brody. A Bateria Breve de Rastreio Cognitivo e o Miniexame do Estado Mental foram utilizados para caracterização da amostra, além da Escala de Depressão Geriátrica. Um total de 61 idosos com idade média de 72,5 anos, predominantemente do sexo feminino (85,2%) e escolaridade média de 11,2 foram avaliados e agrupados de acordo com o seu desempenho no UPSA por meio de uma análise de cluster.

**Resultados:**

A análise revelou três padrões de agrupamento, subdividindo a amostra em subgrupos que se diferenciaram significativamente em termos de idade, escolaridade, cognição global e todas as atividades instrumentais avaliadas pelo UPSA — planejamento, finanças, comunicação, transporte e habilidades domésticas.

**Conclusões:**

O presente estudo foi capaz de identificar a heterogeneidade presente na população idosa entre os diferentes fatores que compõe as AIVDs por meio de uma avaliação baseada em desempenho.

## INTRODUCTION

Factors such as reduced mortality rate and increased life expectancy in the elderly people triggered a new worldwide epidemiological configuration in which the elderly people are estimated as 900 million individuals.^1^ In this scenario, it becomes increasingly relevant to study the heterogeneity that is inherent to this aging process, taking into account the variance of factors that can modulate this process and lead to the formation of different profiles.

Constructs related to functionality function are especially relevant markers in aging and are increasingly explored since they are a particularly relevant health indicator for the elderly people,^2^ given its impact on the quality of life and the consequences they bring to health systems and policies.^3^

Among the aspects that permeate the spectrum of functionality, functional capacity (FC) can be defined as the aptitude and capacity that a given individual has to act in their daily lives independently. Its measurement is done by employing instruments that assess the instrumental activities of daily living (IADL)^4^ ^,^ ^5^ — functioning as a reference to determine the independence of individuals or need for care. An objective way of assessing FC is through performance-based measures such as the University of California, San Diego Performance-Based Skills Assessment (UPSA), which require the performance of tasks in the presence of the examiner.^6^ ^,^ ^7^

Cognitive skills seem to be a relevant aspect of functionality since they are related to performance in IADL.^8^ ^,^ ^9^ At present, additional studies cite the well-established influence of variables such as age and education on levels of independence while others discuss the relationship between FC to other factors such as ethnicity, social class, and distinct health conditions, in addition to gender.^10^ ^,^ ^11^ ^,^ ^12^ ^,^ ^13^

However, despite the diversity of studies discussing FC, it is also possible to observe a tendency to work with the total scores of the instruments, classifying the population as partially/totally dependent or independent based on their final results. Within such a vast population, there is a scarcity of studies that intend to investigate different profiles of functionality in an equally extensive way, seeking to understand the heterogeneity present in the group and classifying them in a nondichotomous way — but instead of it, based on characteristics and patterns observed through similarities and differentiations.

## METHODS

### Sample selection

The sample selection was based on interviews conducted by a qualified psychologist and a doctor member of the research team, during which the following inclusion criteria were assessed: fluency in Portuguese, minimum age of 60 years, and participation in a social program offered by the government of Rio de Janeiro (Casas de Convivência e Lazer para Idosos), whose own requirements for participation are the absence of caregiver and regular frequency on their activities.

The exclusion criteria were also assessed during the interviews, namely, the inability to complete the evaluation battery due to sensory impairment or noncorrectable motor, the presence of major neurocognitive disorder and/or limiting neurological disease, the presence of severe psychiatric disorder, and be illiterate. From the interviews with 63 subjects, 2 of them were excluded for not meeting the selection criteria due to a diagnosed major neurocognitive disorder.

Thus, the final sample consisted of 61 community-active elderly people whose level of independence indicates little or no impairment in the realization of the activities of daily living. Of these, 85.2% were females and had a mean age of 72.48 years (standard deviation [SD]=6.03) and average schooling of 11.16 (SD=4.89).

### Data collection

The subjects were evaluated during three sessions of approximately 60 min, performed by a psychologist and a geriatrician or psychiatrist member of the research team.

### Instruments

Demographic characteristics were collected during an anamnesis interview. Information about the health status of participants (e.g., history of diseases and/or disorders, presence of sensory deficits, cognitive and/or functional complaints, and the medications in use) was collected and evaluated during the medical interview, assisting in the selection of the sample based on the inclusion and exclusion criteria. The assessment of the neuropsychological profile was carried out by a psychologist. 

A performance-based functional assessment was obtained through the application of a translated and adapted version of the UPSA.^7^ ^,^ ^14^ UPSA is characterized as an instrument for the measurement of FC that assesses the performance of individuals in instrumental tasks according to the necessary skills for everyday situations. It is configured as an ecological instrument, seeking to achieve good similarity with the real world through a role-play proposal. Therefore, it evaluates the FC in five areas, namely, planning, money management, communication, use of transportation, and domestic activities,^6^ ^,^ ^7^ ^,^ ^14^ including activities of organization and planning leisure activities, bill payment, using a phone, reading and interpreting city maps and public transport lines, and purchase planning. A brief version of the instrument (UPSA-B),^15^ containing only two of the original five subdomains (i.e., communication and finance), was also adopted in this study. 

Cognitive measures that aided in the characterization of the sample were evaluated by the Brief Cognitive Screening Battery (BCSB), composed of Memory of Figure Test (MFT),^16^ ^,^ ^17^ Verbal Fluency Test (VFT) — animal category,^18^ and Clock Drawing Test (CDT).^16^ ^,^ ^19^ ^,^ ^20^ Besides, the following instruments were also applied: Mini-Mental State Examination (MMSE),^21^ ^,^ ^22^ Geriatric Depression Scale (GDS-15),^23^ ^,^ ^24^ and the self-reported version of the Lawton Instrumental Activities of Daily living Scale.^25^ ^,^ ^26^

## PROCEDURES

### Ethical aspects

This study was part of a larger one involving participants in a social program that was offered by the Special Secretariat for Healthy Aging and Quality of Life of Rio de Janeiro (Casas de Convivência e Lazer para Idosos) and was approved by the Research Ethics Committee (opinion No. 965.264). All study participants signed two copies of informed consent form.

### Statistical analysis

Data analysis was performed using IBM *Statistical Package for the Social Sciences* (SPSS) software version 22 and the Python programming language, including the Scikit-Learn Library. In order to identify the functional profiles at UPSA, grouping the elderly people in a way that maximizes homogeneity within classes, as well as heterogeneity between them, a cluster analysis was performed. The method chosen for this study was the nonhierarchical *k*-means procedure. The algorithm was performed 10,000 times, using the model score extracted from the interaction that resulted in the best performance. The Euclidean distance was used as a distance metric between the centroid and the points in hyperspace. Each measure of functioning related to the instrument was transformed into a Z score before being inserted as an independent variable in the analysis, in order to standardize the results.

A one-way analysis of variance (ANOVA) was performed to test the differences between the groups concerning the scores obtained in UPSA and MMSE, as well as for data regarding delayed memory, age, and education. The effect sizes were calculated using the omega-squared (ω^2^). An analysis of covariance (ANCOVA) was also performed to control the expected effects of age and education on the difference between groups. Finally, the Fisher’s exact χ^2^ test was applied to assess the association between sex and belonging to the cluster.

For all analyses, the main effects were examined with the adoption of a significant F at p<0.05, and the mean values were subjected to *post-hoc* comparison by the Bonferroni test.

For all cognitive variables, the assumption of normality was tested with Shapiro-Wilk tests. In some cases, the distribution was not normal. However, it has been shown that ANOVA can be considered a robust procedure even in cases with deviations from normal.^27^ Besides, in these cases, the use of nonparametric tests such as Kruskal-Wallis presented similar results.

## RESULTS

The characterization of the sample based on the screening instruments that compose the BCSB, as well as the MMSE and scales, are described in Table 1.

**Table 1.** Means and standard deviation for the brief cognitive screening battery, Mini-Mental State Examination, Lawton Instrumental Activities of Daily Living Scale, and Geriatric Depression Scale.

	Nom.	I.M.	Im.M.	Lear.	D.R.M.	Rec.	C.D.T.	V.F.	ME30	IADL	GDS
Mean	9.9	5.7	8.3	8.8	8.2	10.0	6.2	18	26.4	20	2.8
SD	0.2	1.4	1.4	1.4	1.6	0.2	2.3	7.1	2.8	1.1	2.8

SD: standard deviation; BCSB: brief cognitive screening battery, Nom.: nomination; I.M.: incidental memory; Im.M.: immediate memory; Lear.: learning; D.R.M.: delayed recall memory test; Rec.: recognition; C.D.T.: clock drawing test; V.F.: verbal fluency test; ME30: MMSE-30; IADL: Lawton Instrumental Activities of Daily Living Scale; GDS: Geriatric Depression Scale.

From this sample, scenarios were evaluated considering 2–8 clusters (K) in order to determine the K value capable of maximizing the homogeneity of the objects within the groups and the heterogeneity between them. A 3-group solution for the *k*-means algorithm resulted in qualitatively and quantitatively better-distributed clusters, having been selected. For this K, a total of three iterations were executed, promoting changes in the centers of each cluster. The characterization of the clusters and their final centers are described in Table 2.

**Table 2.** Clusters’ final centers.

UPSA (Z score)	Cluster 1 (n=23)	Cluster 2 (n=16)	Cluster 3 (n=22)
Planning domain	-0.04	-0.90	0.70
Finances domain	-0.07	-1.05	0.84
Communication domain	-0.38	-0.57	0.81
Transportation domain	0.14	-0.94	0.53
Household chores domain	0.32	-1.07	0.44
UPSA — brief	-0.24	-0.99	0.98
UPSA — total score	-0.08	-1.28	1.02

PSA: University of California, San Diego Performance-Based Skills Assessment.

The characterization of each cluster in terms of schooling, age, global cognition, and memory is described in Table 3.

**Table 3.** Sample characterization.

Cluster		Schooling	Age	MMSE	D.R.M.
1	Mean	11.91	73	27.22	8.33
	Standard deviation	4.74	5.09	1.24	1.53
2	Mean	6.94	75	23.56	8.38
	Standard deviation	4.19	7.08	3.24	1.89
3	Mean	13.45	70.09	27.73	7.90
	Standard deviation	3.54	5.47	2.16	1.29
Total	Mean	11.16	72.48	26.44	8.19
	Standard deviation	4.89	6.03	2.81	1.55

MMSE: Mini-Mental State Examination; D.R.M.: delayed recall memory test.

ANOVA showed that there is a significant effect of the group on the following variables: education [F=(2, 58)=11.75; p<0.001; ω^2^=0.51], age [F=(2, 58)=3.47; p=0.038; ω^2^=0.27], global cognition [F=(2, 58)=18.270; p<0.001; ω²=0.60], UPSA subdomains: planning [F=(2, 58)=19.06; p<0.001; ω^2^=0.61], finance [F=(2, 58)=36.23; p<0.001; ω^2^=0.73], communication [F=(2, 58)=17.93; p<0.001; ω^2^=0.60], transport [F=(2, 58)=15.31; p<0.001; ω^2^=0.57], and household chores [F=(2, 58)=20.50; p<0.001; ω^2^=0.63], UPSA shortened version [F=(2, 58)=50.42; p<0.001; ω^2^=0.79], and full version [F=(2, 58)=135.21; p<0.001; ω^2^=0.90]. In turn, the memory variable measured by the delayed recall memory test, did not show any significant difference between the groups [F=(2, 58)=0.54; p=0.58]. ANCOVA was also carried out to control the effects of the influence of age and education on the functional performance variance between the groups. The comparisons can be found in Table 4.

**Table 4.** Multiple comparisons

	Clusters			Sig. ANOVA	Sig. ANCOVA
Schooling	1	2	–	0.002	–
	1	–	3	0.670	–
	–	2	3	<0.001	–
Age	1	2	–	0.881	–
	1	–	3	0.294	–
	–	2	3	0.038	–
MMSE	1	2	–	<0.001	0.003
	1	–	3	1	1
	–	2	3	<0.001	0.007
Delayed recall memory test	1	2	–	1	1
	1	–	3	1	1
	–	2	3	1	0.758
UPSA Planning	1	2	–	<0.001	0.005
	1	–	3	0.008	0.006
	–	2	3	<0.001	<0.001
UPSA Finances	1	2	–	<0.001	0.008
	1	–	3	<0.001	<0.001
	–	2	3	<0.001	<0.001
UPSA Communication	1	2	–	1	1
	1	–	3	<0.001	<0.001
	–	2	3	<0.001	0.007
UPSA Transportation	1	2	–	<0.001	0.043
	1	–	3	0.345	1
	–	2	3	<0.001	0.015
UPSA Household Chores	1	2	–	<0.001	<0.001
	1	–	3	1	1
	–	2	3	<0.001	0.001
UPSA Brief	1	2	–	<0.001	0.084
	1	–	3	<0.001	<0.001
	–	2	3	<0.001	<0.001
UPSA Total Score	1	2	–	<0.001	<0.001
	1	–	3	<0.001	<0.001
	–	2	3	<0.001	<0.001

ANOVA: analysis of variance; ANCOVA: analysis of covariance; UPSA: University of California, San Diego Performance-Based Skills Assessment; MMSE: Mini-Mental State Examination. For values described in bold, the mean difference is significant at the 0.05 level.

Fisher’s exact χ^2^analysis showed that there is an association between gender and belonging to a certain cluster [χ^2^(2)=7.035; p<0.05]. The *post-hoc* corrected by Bonferroni pointed to a significant difference in the distribution by gender only in Cluster 3, composed of 22 women and 0 men (expected n=18.8 women and 3.2 men; adjusted residuals=|2.44|). Clusters 1 and 2 maintained the gender distribution within the expected by the model, also considering the adjusted residuals.

## DISCUSSION

The results confirmed that it is a group whose cognitive and functional characteristics are within the expected for a population of the elderly people who attend the social program in Rio de Janeiro.^28^ Besides, the average score obtained on the geriatric depression scale is similar to that expected for this population, being below the cutoff points most strongly recommended in the literature for detecting depression.^29^

Thus, the sample was divided into three subgroups according to the performance obtained in a variety of activities of daily living that constitute the UPSA. The final centers indicate the groups maintaining intra-cluster homogeneity, since we can observe a tendency to perform with little variability.

In group 1, the total Z score (Z=-0.08) indicates performance and FC close to the sample mean. Group 3 also did not show any functional impairment, obtaining a Z score of 1.02 for the total UPSA, indicating a performance, in fact, slightly above average.

In contrast, group 2 has a substantially different profile. The value of Z score for the total score on the instrument is -1.28, indicating an overall FC with a slight impairment. The functional impairment that characterizes this group is also accompanied by a lower global cognitive level compared with other groups (MMSE mean score=23.56, SD=3.24), although no differences related to memory were detected.

The results regarding the variable late memory recovery, a component of the figure memory test, provide reflections on the cognitive functions of samples. Although the results presented regarding the MMSE have been controlled in terms of age and education, the TMF stands out because it is an instrument that in itself has little influence on the education variable.^30^ ^,^ ^31^ ^,^ ^32^ According to the ANOVA, however, the three clusters do not show any difference concerning the score results. Thus, we understand that although the groupings may differ in terms of global cognition, this difference does not appear to be due to mnemonic changes.

This neuropsychological profile seems to agree with the literature when characterizing individuals with a nonamnestic type of mild cognitive disorder (MCI) — defined as a clinical condition in which the individual has a slight decline in any cognitive domain other than the memory, without meeting the clinical diagnostic criteria for dementia.^33^

The studies that point to a cutoff point on the MMSE for the diagnosis of MCI vary in their findings due to methodological differences, the version used of the instrument, and, above all, due to the diversity and variation in the characteristics of the studied samples, since age and education are the factors of strong influence on the final score of a person,^16^ ^,^ ^34^ making it difficult to compare research results (Melo & Barbosa, 2015). At present, the studies are consistent in pointing to a significant difference between the scores of individuals with MCI and controls.^35^

Also according to the DSM-V,^33^ the cognitive deficits present in this clinical condition may be sufficient as a need for more effort, compensatory strategies, or accommodation during the realization of instrumental activities. Thus, although the basic activities of daily living must be preserved, complex instrumental activities can be minimally compromised.^36^

Particularly, the performance in financial activity has been gaining attention, since it seems to be able to differentiate control individuals from those diagnosed with MCI. According to the literature, patients with MCI perform significantly worse than controls in financial activities, reflecting a longer time to complete tasks and a greater tendency to errors, less financial conceptual knowledge, and a more prominent difficulty in managing bank statements and paying bills.^37^ These differences could be justified due to the high attentional and executive demands that are inherent to the execution of such activities.^37^

Although all the component activities of the instrument differ between clusters 2 and 3 (referring to individuals with worse and better performances) when compared with ANOVA, only two of them stand out for being significant in the three levels of comparison: “planning” and “finance”— this being the largest effect size. Thus, once again, the performance in “finance” stands out as a relevant clinical feature to be analyzed in this context in conjunction with the general FC.

Among all the skills assessed, communication activity, in turn, was the only one that was not significant between clusters 2 and 1, indicating that there is little decline in this activity when comparing a group of average performance with a group with slight impairment. Despite the existence of studies that point to impaired communication skills in individuals with cognitive decline, most of them focus on the domains of expressive and receptive communication, including verbal fluency, motor speech production, sentence comprehension, receptive speech, and reading comprehension.^38^ Such aspects are different from those addressed in the communication assessment of UPSA, which focuses specifically on the successful use of a telephone instrument. Besides, these studies use cognitive instruments instead of ecological and functional instruments, leading to a discrepancy in the methodology.

Another point to be addressed is the fact that there is a heterogeneity of performance between the functionally preserved groups (1 and 3), although this difference is not reflected in terms of MMSE scores, delayed memory, age, or education. On the contrary, it implies the existence of an elderly profile similar to group 1 in terms of demographic characteristics, but with significantly superior performance in planning, finance, and communication activities, leading to a higher overall functional profile.

Finally, as the activities “transportation” and “household chores” remained close to the average, with no significant differences between clusters 1 and 3, we can assume that there are activities that vary less in functionally preserved individuals but decline significantly when compared with a group, such as group 2, with a slight functional impairment.

The covariance analyzes performed showed, in general, results similar to ANOVA, indicating that the results found in terms of differences in functionality do not appear to be due to differences in age and education. 

The comparison between clusters 1 and 2 within UPSA-B seemed to be the only exception and can be explained by the combination of the decrease in the mean difference in scores in both variables that constitutes the short version. The version, however, remained significant when controlling only the age variable, indicating that schooling may be a factor of greater differential relevance in this context. This finding can be confirmed by the comparison between the effect sizes. Also, it is in agreement with the literature, which indicates that in terms of comparative analysis between groups, the variable education seems to be the most relevant in most cases.^39^

In terms of gender-related findings, the existence of a different performance in IADLs is discussed in the literature, associating women with a higher level of fragility and worse performance in activities.^12^ ^,^ ^13^ The findings of this study, however, contradict this hypothesis by pointing to a homogeneous distribution between female and male individuals in the worst-performing cluster, while also pointing to the existence of a significant difference between genders when analyzing the cluster with better functional performance, composed of only women.

However, it is necessary to point out that there is a great disparity between the number of women (85.2%) and men (14.8%) in the sample, which may justify such a discrepancy between this study and the literature. Such a disparity, however, is not uncommon, and in a study carried out with a similar sample, the percentage of women was also significantly higher, making up 89% of the sample.^28^ In reality, this characterization seems to follow the national trend of feminization of the elderly population.^40^

It is not the intention of this study, given its transversal nature, to establish causal relationships between variables — just to verify the difference between the existing functional profiles and to characterize their subsamples. At present, the results presented in this study should not be generalized to the different populations, since it is a sample from the community living in Rio de Janeiro, characterized by their own singularities. Another limitation of this study is the sample size, so that replication with a more representative higher n is recommended.

Also, the psychiatric/geriatric interview and the instruments used were designed to identify individuals who met the criteria for inclusion in this study, since the objective was the functional characterization of an independent or partially independent community sample. A more detailed cognitive characterization, though, may be a suggestion for future studies.

At present, the use of an instrument such as the MMSE and BCSB in this study is valid given the objective of global cognitive characterization of the sample. However, the association of this with instruments of greater sensitivity for frontal impairments, such as those related to executive functions, would resu lt in a more complete cognitive profile, especially considering that the functional changes detected in this study may be related, in a greater degree, to other cognitive variables besides the memory.

Despite its limitations, this study was successful in its proposal to explore the patterns of IADL between community-dwelling older adults.
